# Transcriptional effects of 1,25 dihydroxyvitamin D_3_ physiological and supra-physiological concentrations in breast cancer organotypic culture

**DOI:** 10.1186/1471-2407-13-119

**Published:** 2013-03-15

**Authors:** Cintia Milani, Maria Lucia Hirata Katayama, Eduardo Carneiro de Lyra, JoEllen Welsh, Laura Tojeiro Campos, M Mitzi Brentani, Maria do Socorro Maciel, Rosimeire Aparecida Roela, Paulo Roberto del Valle, João Carlos Guedes Sampaio Góes, Suely Nonogaki, Rodrigo Esaki Tamura, Maria Aparecida Azevedo Koike Folgueira

**Affiliations:** 1 Disciplina de Oncologia, LIM24, Departamento de Radiologia e Oncologia, Faculdade de Medicina da Universidade de São Paulo, Av. Dr. Arnaldo 455,Sala 4124, São Paulo, SP, 01246-903, Brazil; 2 Instituto Brasileiro de Controle do Câncer, IBCC, São Paulo, Brazil; 3 Department of Environmental Health Sciences, School of Public Health, University at Albany, Rensselaer, NY, 12144, USA; 4 Hospital do Câncer A. C. Camargo, São Paulo, Brazil; 5 Instituto Adolfo Lutz, Centro de Patologia, Núcleo de Patologia Quantitativa, São Paulo, Brazil; 6 Post-doctoral fellow, Viral Vectors Laboratory, Instituto do Câncer do Estado de São Paulo, ICESP, São Paulo, Brazil

**Keywords:** Breast cancer, Calcitriol, Gene expression, Organotypic culture

## Abstract

**Background:**

Vitamin D transcriptional effects were linked to tumor growth control, however, the hormone targets were determined in cell cultures exposed to supra physiological concentrations of 1,25(OH)_2_D_3_ (50-100nM). Our aim was to evaluate the transcriptional effects of 1,25(OH)_2_D_3_ in a more physiological model of breast cancer, consisting of fresh tumor slices exposed to 1,25(OH)_2_D_3_ at concentrations that can be attained *in vivo*.

**Methods:**

Tumor samples from post-menopausal breast cancer patients were sliced and cultured for 24 hours with or without 1,25(OH)_2_D_3_ 0.5nM or 100nM. Gene expression was analyzed by microarray (SAM paired analysis, FDR≤0.1) or RT-qPCR (p≤0.05, Friedman/Wilcoxon test). Expression of candidate genes was then evaluated in mammary epithelial/breast cancer lineages and cancer associated fibroblasts (CAFs), exposed or not to 1,25(OH)_2_D_3_ 0.5nM, using RT-qPCR, western blot or immunocytochemistry.

**Results:**

1,25(OH)_2_D_3_ 0.5nM or 100nM effects were evaluated in five tumor samples by microarray and seven and 136 genes, respectively, were up-regulated. There was an enrichment of genes containing transcription factor binding sites for the vitamin D receptor (VDR) in samples exposed to 1,25(OH)_2_D_3_ near physiological concentration. Genes up-modulated by both 1,25(OH)_2_D_3_ concentrations were *CYP24A1, DPP4, CA2*, *EFTUD1, TKTL1, KCNK3*. Expression of candidate genes was subsequently evaluated in another 16 samples by RT-qPCR and up-regulation of *CYP24A1, DPP4* and *CA2* by 1,25(OH)_2_D_3_ was confirmed. To evaluate whether the transcripitonal targets of 1,25(OH)_2_D_3_ 0.5nM were restricted to the epithelial or stromal compartments, gene expression was examined in HB4A, C5.4, SKBR3, MDA-MB231, MCF-7 lineages and CAFs, using RT-qPCR. In epithelial cells, there was a clear induction of *CYP24A1*, *CA2*, *CD14* and *IL1RL1.* In fibroblasts, in addition to *CYP24A1* induction, there was a trend towards up-regulation of *CA2, IL1RL1*, and *DPP4*. A higher protein expression of CD14 in epithelial cells and CA2 and DPP4 in CAFs exposed to 1,25(OH)_2_D_3_ 0.5nM was detected.

**Conclusions:**

In breast cancer specimens a short period of 1,25(OH)_2_D_3_ exposure at near physiological concentration modestly activates the hormone transcriptional pathway. Induction of *CYP24A1*, *CA2, DPP4, IL1RL1* expression appears to reflect 1,25(OH)_2_D_3_ effects in epithelial as well as stromal cells, however, induction of *CD14* expression is likely restricted to the epithelial compartment.

## Background

Epidemiological data indicates higher incidence and mortality rates from breast cancer in low latitude regions. Among the mechanisms suggested for a relationship between sunlight and cancer is the genesis of vitamin D in the skin, resulting from the UV light action. In accordance with this hypothesis, there is evidence that lower 25(OH)D_3_[1-5] and 1,25(OH)_2_D_3_[6,7] serum concentrations are encountered in patients with breast cancer, as compared with women without cancer, as well as in patients with advanced or metastatic disease in comparison with those with early-stage disease [8,9]. In addition, 25(OH)D_3_ deficiency at diagnosis was related with poor prognosis, evaluated as metastasis-free and overall survival [10].

In human breast xenografts established in immunossupressed mice 1,25(OH)_2_D_3_ exerts growth inhibitory effects, and in mouse mammary organ culture exposed to chemical carcinogens, both 25(OH)D_3_ and 1,25(OH)_2_D_3_ mediate preventive effects [11-13]. However, the chemopreventive effect of vitamin D is still controversial, as supplementation trials on vitamin D_3_ and colon or breast cancer incidence have been inconsistent [14,15]. One critical issue is that the appropriate supplementation dose for cancer prevention trials was not well established [16]. On the other hand, clinical studies point to a clinical benefit for 1,25(OH)_2_D_3_ (or analogues) alone or in combination with chemotherapy in the treatment of hormone refractory prostate cancer and breast cancer skin lesions [17,18]. However, concerns about hypercalcemic side effects limit the dose of 1,25(OH)_2_D_3_ (or analogues) that can be safely administered *in vivo*.

Phase I clinical studies indicate that subcutaneous doses of calcitriol given every other day result in peak 1,25(OH)_2_D_3_ serum concentration of 0.25-0.75 nM [19] while weekly pulses of oral calcitriol allow higher dose administration and peak serum concentrations of 1–15 nM [20]. Although these vitamin D concentrations represent about 1.3-83 times the upper limit of physiologic serum levels, they are well below the concentrations (10-100nM) typically used to investigate hormone actions in cell culture studies. At these concentrations, 1,25(OH)_2_D_3_ exerts antiproliferative and pro apoptotic effects [21] and modulates angiogenesis [22,23], invasion and metastasis [24,25]. Among the downstream targets of the hormone are cyclin dependent kinase inhibitors as p21^WAF1/CIP1^ and p27^KIP1^; growth factors, receptors and associated proteins as TGFβ, TGFβ receptors and insulin-like growth factor binding protein-3 (IGFBP-3) [26-31]. In addition, gene expression profiling of breast cancer cell lines MCF7 and MDA-MB-231 have identified many potential 1,25(OH)_2_D_3_ target genes, [24] but again, these studies were conducted with supra physiological concentrations of calcitriol (50-100nM). Furthermore, experiments in cell lines do not reflect the complex array of interactions among malignant and stromal cells, secreted factors and extracellular matrix proteins taking place in the tumor microenvironment, which also modulate the hormone actions.

Although the majority of human breast cancers express vitamin D receptors (VDR) [7,32,33], there have been no demonstrations that 1,25(OH)_2_D_3_ modulates gene expression in human breast cancer samples. To address this research gap, a physiologically relevant *in vitro* model to study 1,25(OH)_2_D_3_ actions, represented by short term culture of fresh breast cancer tissue slices, which maintain the epithelial mesenchymal relationship and preserve tissue morphology and proliferation rate, was established [25,34,35]. With this organotypic culture system the transcriptional effects of 1,25(OH)_2_D_3_ at 0.5nM, a concentration that can be safely attained *in vivo,* and 100nM, the concentration typically used in cell culture studies, was compared. In addition, mammary cell lines and fibroblasts obtained from breast cancer samples were used to validate transcriptional targets of 1,25(OH)_2_D_3_ in epithelial and stromal cell types. Cancer associated fibroblasts (CAF) are interactive cells that infiltrate tumor specimens, influencing their behavior [36-38], which are also potential targets of the hormone. Although VDRs have been detected in fibroblasts obtained from prostate and breast tumors, few studies have compared 1,25(OH)_2_D_3_ mediated genomic effects in epithelial and stromal cells [39,40]. The present study indicates that physiologically relevant concentrations of 1,25(OH)_2_D_3_ may influence gene expression in breast tumor slices cultured *ex vivo*, and that regulation of target genes likely occurs in both epithelial and stromal compartments of the tumor.

## Methods

### Patients

Post-menopausal breast cancer patients clinical stages I-III were invited to take part in the study. This protocol was carried out in compliance with the Helsinki Declaration and was approved by the Institutional Ethics Committee (Comitê de Ética do Instituto Brasileiro de Controle do Câncer, protocol number 108/2006/7; Comitê de Ética em Pesquisa do Hospital das Clínicas da Faculdade de Medicina da Universidade de São Paulo, protocol number 626/06; Comitê de Ética do Hospital do Câncer A. C. Camargo, protocol number 1131/08). A written informed consent was signed by all participants. Twenty one patients were prospectively accrued at Instituto Brasileiro de Controle do Câncer and Hospital do Câncer A. C. Camargo, São Paulo, from August 2007 to September 2009. Characteristics of these patients are described on Table 1.

**Table 1:** Characteristics of patients

	***Training group (n=5)***	***Validation group (n=16)***	***p***
Median age	70 (56–76)	56.5 (49–72)	0.068
CS III	3 (60%)	5 (33%)	0.347
N(+)	3 (60%)	9 (60%)	1.000
IDC	5 (100%)	10 (62%)	0.262
ER(+)	2 (40%)	11 (69%)	0.325
PR(+)	3 (60%)	9 (56%)	1.000
HER2(+)	2 (40%)	5 (31%)	1.000

Tumor samples collected for tissue slices culture. Age in years; CS III: clinical stage III, AJCC 2002; IDC: invasive ductal carcinoma; N(+): lymph node positive involvement; ER: estrogen receptor immunoexpression; PR: progesterone receptor immunoexpression; HER2 immunoexpression (+): 3+/3+; (-): negative; (+): positive. p: Fisher exact test or Mann Whitney test.

### Tissue slice preparation and treatment

Tumor fragments were obtained immediately after tumor resection by the pathologist, who selected an involved area for this study. Fragments were placed into culture medium (RPMI 1640 with antibiotics and fungicide) and tissue slices were prepared using the Krumdieck tissue slicing system (Alabama Research and Development Corporation, Birmingham, AL, USA). Fragment thickness varied between 400–800 μm. Slices were cultured for 24 hours in 6-well plates (1 slice/well; 1–3 slices per treatment) containing 2 mL of culture media, RPMI supplemented with 10% v/v FBS, antibiotics and 0.001% ethanol (vehicle) or 1,25(OH)_2_D_3_ (Calbiochem, Darmstadt, Germany) 0.5nM or 100nM (from now on called physiological and supra-physiological concentrations, respectively). One slice of each sample was processed by FFPE and hematoxilin-eosin stained slides revealed that tumor samples contained > 50% malignant cells.

### Fibroblasts primary culture

Primary fibroblast culture was established from tumor samples obtained from another five post-menopausal patients, diagnosed with invasive ductal carcinoma (histological grades II or III, three of them hormone receptor positive). Tumor samples were cut into small pieces and fibroblast primary culture was established through the explant methodology. After three cell passages, mesenchymal origin of the cells was confirmed by their spindle cell morphology and positive expression of vimentin [mouse anti human vimentin monoclonal antibody, clone Vim 3b4 (1:200); DAKO Corporation, Carpinteria, CA, USA] and alpha smooth muscle actin [(mouse monoclonal antibody anti human alpha smooth muscle actin, clone 1A4 (1:50); R&D Systems] and negative expression of cytokeratin [mouse monoclonal antibody anti human cytokeratin clone AE1/AE3 (1:100); DAKO] by immunocytochemistry (data not shown). Fibroblasts were then exposed to 1,25(OH)_2_D_3_ (Calbiochem, Darmstadt, Germany) 0.5nM or vehicle for 24 hours and after RNA extraction, RT-qPCR was performed to evaluate expression of candidate genes.

### Culture of mammary epithelial cell lines

HB4A (normal mammary epithelial cell line) and C5.2a (HB4A transfected with HER2), both donated by Drs. Mike O’Hare and Alan Mackay, Ludwig Institute for Cancer Research, London, UK; SKBR3: breast cancer cell line overexpressing HER2; MDA MB-231: breast cancer cell line triple negative; and MCF-7: breast cancer cell line ER(+), acquired from American Type Culture Colection (Manassas, Virginia, USA), were cultured in RPMI-1640 supplemented with 10% fetal calf serum (FCS). After 24 hours, medium was replaced and 1,25(OH)_2_D_3_ 0.5 nM (treated cells) or ethanol (control cells) was added. After 24 hs of treatment, total RNA was isolated using Trizol reagent and used in RT-qPCR.

### RNA extraction and microarray hybridization

Tumor specimens were pulverized (Bio-Pulverizer™ BioSpec Products Inc., Oklahoma, USA) under liquid nitrogen and total RNA was isolated using RNeasy kit (Qiagen, Valencia, CA, USA), according to the manufacturer’s protocol. RNA integrity was verified in a Bioanalyzer 2100 (Agilent Technologies, Santa Clara, CA, USA) and samples with RNA integrity number ≥ 6.6 were analyzed. Beginning with 100 ng total RNA, a two-round linear amplification was carried out, according to Affymetrix protocol (Two Cycle Target Labeling Kit, Affymetrix, Santa Clara, CA, USA). Afterwards, biotin-labeled cRNA was synthesized from double strand cDNA, using IVT labeling kit (Affymetrix) and 20 μg of biotinylated fragmented aRNA was hybridized onto Human Genome U133 Plus 2.0 GeneChip (Affymetrix

Hybridized arrays were scanned using Affymetrix GeneChip Scanner 3000 and after visual inspection, images were subjected to Affymetrix GeneChip Operating Software (GCOS) analysis to generate report files for quality control. Data normalization was performed using the Robust Multi-Array Average (RMA). Samples were categorized according to treatment in three groups: 1,25(OH)_2_D_3_ 0.5nM, 1,25(OH)_2_D_3_ 100nM and control. To establish a differential gene expression profile between vitamin D treated and untreated samples, SAM two class paired, provided on MEV (MultiExperiment Viewer – Boston, MA, USA) was used, after selecting 50% of the genes with the highest standard deviation. False discovery ratio (FDR) ≤0.10 was considered significant. In addition, results obtained with FDR≤0.01 are presented. Unsupervised hierarchical clustering based on Euclidean distance and average linkage was used to verify association patterns. The reliability of the clustering was assessed by the Bootstrap technique. Raw data complying with MIAME format was deposited at the Gene Expression Omnibus (GEO) data repository (http://www.ncbi.nlm.nih.gov/geo/query/acc.cgi?acc=GSE27220) accession number GSE27220.

To explore functional enrichment associated with calcitriol treatment based on Ontologies (GO, Pathway), Regulome (TFBS, transcription factor binding site) Pharmacome (Drug-gene associations) among other features, differentially expressed genes were subject to subsequent analysis using ToppFun, available on ToppGene Suite (http://toppgene.cchmc.org/enrichment.jsp) and were considered significant if *P* < 0.05 [41].

Gene set enrichment analysis (GSEA) method was used to identify whether predefined gene sets might associate with gene expression differences between phenotypes. In this pairwise comparison, all genes are ranked based on signal-to-noise ratio and the alternative hypothesis that rank ordering of distinct pathway members is associated with a specific phenotype is tested [42]. This methodology makes it possible to detect situations where all genes in a predefined set change in a small but coordinated way. FDR<0.10 was considered significant.

### Real time RT-PCR

Reverse transcription was performed with random primers and Superscript III (Invitrogen Corporation, Carlsbad, CA, USA). Quantitative PCR (qPCR) was carried out using specific primers (Additional file 1: Table S1) and SYBR-green I (Sigma, St. Louis, MO, USA) in a Rotor-gene system (Corbett Research, Mortlake, Australia). Relative expression of target genes was calculated as 2^-ΔΔCT^, using *GAPDH* or *ACTB* as internal control (as indicated) and the average value of the target gene in control samples, as reference level.

### Western blot

Protein lysates from cell lines were made using RIPA buffer (1% NP-40, 0.1% SDS, 0.5% Sodium Deoxycholate in 1 × PBS) supplemented with complete mini protease inhibitor cocktail tablets (Roche; cat 04693124001). Afterwards, 50 μg of protein was subjected to SDS-PAGE and transferred to Hybond ECL membrane (GE Lifesciences), which was probed with the following primary antibodies overnight at 4°C: CD26 (DPP4, clone H-270, rabbit polyclonal antibody, 1:500; Santa Cruz Biotechonology Inc. Santa Cruz, CA, USA); CD14 (clone M305, sc9150, rabbit polyclonal antibody 1:500; Santa Cruz); β-actin (monoclonal Anti-β-Actin antibody produced in mouse, clone AC-15, ascites fluid, A5441, 1:2000, Sigma-Aldrich) and then with appropriate secondary antibodies (170–6515 Goat Anti-Rabbit Ig-G (H+L) HRP conjugate; 170–6516 Goat Anti-Mouse Ig-G (H+L) HRP conjugate; Bio-Rad.). Protein expression was detected with ECL Plus Western Blotting Detection Reagents (GE Lifesciences) in a ImageQuant LAS 4000 (GE Healthcare).

### Immunocytochemistry

Fibroblasts were grown on coverslips in the absence or presence of 1,25(OH)_2_D_3_ 0.5nM for 24 hours. Samples were fixed in 4% paraformaldehyde and permeabilized with 0.5% Triton X-100/PBS, in case of intracellular targets. Blocking of unspecific binding was performed with 2% BSA/PBS. Afterwards, cells were incubated with the primary antibody (CD26, clone H-270 rabbit polyclonal antibody, anti-DPP4, 1:200, Santa Cruz; CA II, clone G-2, sc-48351 mouse monoclonal antibody, 1:100, Santa Cruz) overnight in humid chamber at 4°C and then with the secondary antibody conjugated with Alexa Fluor 488 (1:700, species specific: goat anti-rabbit IgG, n° A11008; goat anti-mouse IgG n° A11001; Molecular Probes) for 1 h at room temperature in the dark. DAPI was added for nuclear staining. Images were acquired in a Olympus fluorescence microscope DX-5AI, using an Image Pro-PLUS 6,0 software.

### Immunohystochemistry

Breast cancer slices from seven patients (six samples cultured in the absence (control) or presence of 1,25(OH)_2_D_3_ 100nM and one sample cultured in the presence of 1,25(OH)_2_D_3_ 0.5nM) were available for analysis (from the patients described in Table 1). Sections of 3 μm thickness were cut from paraffin blocks and antigen retrieval was carried out in 10 mM citrate buffer at pH 6.0 in humid heat under pressure cooker. Staining with the following specific antibody took place overnight at 4°C: CD14, clone M-305, (sc-9150Santa Cruz Biotechnology) rabbit polyclonal IgG, 1:800. Reaction was revealed with Novolink Polymer Detection Systems (Leica Biosystems, Newcastle, UK, cat: RE7280-k), followed by analysis in a Olympus fluorescence microscope DX-5AI (40x objective) and acquisition with an Image Pro-PLUS 6,0 software.

### Detection of soluble CD14 (sCD14) in culture medium of tumor samples

Tumor slices from another four post-menopausal patients (median age 56 years) diagnosed with invasive ductal carcinoma clinical stages I-II, HER2 negative and hormone receptor positive (except for one tumor triple negative) were cultured with or without 1,25(OH)_2_D_3_ 0.5nM or 100nM for 24 hours and 100 μL of the conditioned medium was used for soluble CD14 (sCD14) quantitative determination, through an enzyme-linked immunosorbent assay (Quantikine ELISA Human sCD14 Immunoassay, R&D Systems, Minneapolis, MN, USA). For every sample, two analyses on the same plate were carried out and the mean value was used.

### Statistics

Kolmogorov-Smirnov test was applied to check for normality of the data, followed by parametric or non-parametric tests, as appropriate. To detect an association between variables, Pearson chi-square or Fisher exact tests were used. A two-tailed *p* value ≤ 0.05 was considered significant. Analysis was undertaken using Instat (GraphPad Software, Inc., La Jolla, CA, USA) or SPSS (Chicago, IL, USA).

## Results

### Patients characteristics

Twenty one post-menopausal patients with breast cancer clinical stages I-III were included in this study. Samples from five patients were analyzed in a training group, using microarray, and from another 16 patients were analyzed in a validation group, using RT-qPCR. There were no differences between groups concerning age, clinical stage, lymph node involvement; ductal histology; ER, PR and HER2 immunoexpression (Table 1).

### Vitamin D transcriptional effects in breast cancer slices

**Figure 1:**
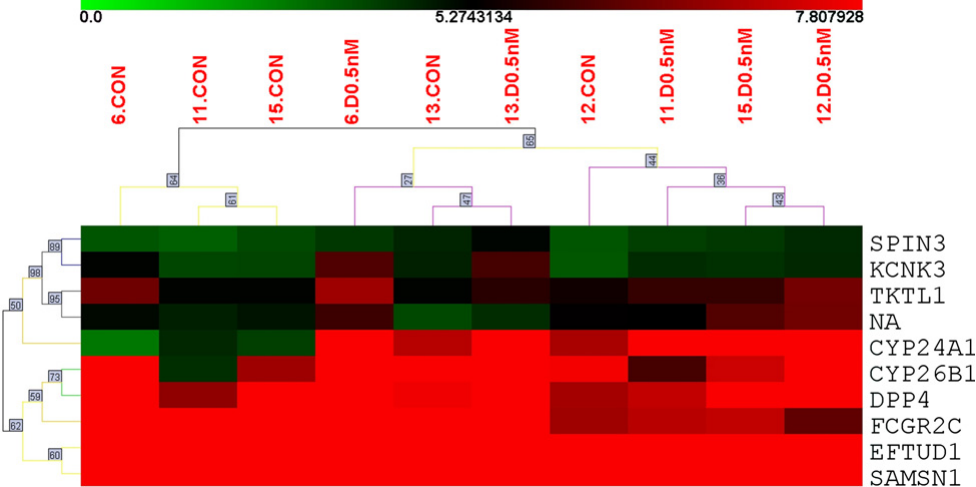
**Unsupervised hierarchical clustering of breast cancer slices exposed to vehicle or 0.5nM 1,25(OH)**_**2**_**D**_**3**_**.** Five tumor samples (6, 11, 12, 13, 15) were cultured with or without 0.5nM 1,25(OH)_2_D_3_ for 24 h. Gene expression was evaluated by microarray and analysis performed using SAM paired analysis (FDR ≤ 0.1). The upper colored bar and values indicate gene expression in target samples (i.e., red, more expressed and green, less expressed). Colored lines of the dendrogram and numbers stand for the support for each clustering, black and gray, more reliable; yellow and red, less reliable. Nine genes were differentially expressed (plus one probe without a gene name, NA). Two branches are identified, one of them including three control samples.

At first, the transcriptional effects of 0.5 nM 1,25(OH)_2_D_3_ vs control in breast cancer slices were compared, using SAM paired analysis (FDR ≤ 0.1). As shown in Table 2, seven genes were up-regulated and two genes were down-regulated in tumor slices exposed to 0.5nM 1,25(OH)_2_D_3_ for 24 h. Enrichment of genes involved in vitamin metabolic process (*TKTL1, CYP24A1, CYP26B1*) was observed. Unsupervised clustering of the differentially expressed genes identified two branches, however there was no aggregation of samples according to 1,25(OH)_2_D_3._ treatment (Figure 1). At a more stringent FDR level (≤ 0.01), only five (*CYP24A1, DPP4, EFTUD1, FCGR2C, SAMSN1*) genes were differentially expressed.

**Table 2:** **Genes differentially modulated in breast tumor slices incubated with 0.5nM 1,25(OH)**_**2**_**D**_**3**_

***Gene title***	***Gene symbol***	***FC***
cytochrome P450, family 24, subfamily A, polypeptide 1	**CYP24A1**	7.54
dipeptidyl-peptidase 4 (CD26, adenosine deaminase complexing protein 2)	**DPP4**	2.01
cytochrome P450, family 26, subfamily B, polypeptide 1	CYP26B1	1.94
potassium channel, subfamily K, member 3	KCNK3	1.92
transketolase-like 1	TKTL1	1.55
spindlin family, member 3	SPIN3	1.55
elongation factor Tu GTP binding domain containing 1	**EFTUD1**	1.49
Fc fragment of IgG, low affinity IIc, receptor for (CD32)	**FCGR2C**	-1.61
SAM domain, SH3 domain and nuclear localization signals 1	**SAMSN1**	-1.79

SAM paired analysis FDR ≤ 0.10 (FDR ≤ 0.01, genes marked in bold). FC: Fold change of the ratio. (-): lower expression in 1,25(OH)_2_D_3_ treated (as compared with untreated) samples.

Using GSEA (motif, transcription factors), to compare samples treated with 0.5nM 1,25(OH)_2_D_3_ treated and untreated, only one gene set was enriched at FDR ≤ 0.1, namely DR3, comprising genes containing a motif for vitamin D receptor (VDR) around the transcription start site (Additional file 2: Table S2).

Next, the effects of a 24 h exposure to 1,25(OH)_2_D_3_ 100nM was evaluated in this model system. Using FDR cut offs of ≤ 0.1 and ≤ 0.01, 196 (136 more and 60 less expressed in treated samples) and 30 (28 more and 2 less expressed in treated samples) candidate target genes were found, respectively (Additional file 3: Table S3). Up-regulated genes were involved in vitamin metabolic process, regulation of leukocyte mediated immunity and positive regulation of alpha-beta T cell activation. In addition, some genes were associated with calcitriol and TGF-beta signaling pathway (Table 3). There was no separation of treated and untreated samples, upon unsupervised hierarchical cluster analysis, and paired tumors co-aggregated in the same branch (Additional file 4: Figure S1).

**Table 3:** **Functional categories of genes up-regulated in breast tumor slices incubated in 100 nM 1,25(OH)**_**2**_**D**_**3**_

	***Genes up-regulated in VD3 100nM treated samples***
***Biological process***	
Response to external stimulus	*TGFBR2, LXN, THBD,PTEN, PTGER3, HBEGF, CYP24A1, ACVRL1, CCL19, FOXF1, FYN, OSM, CD28, CD14, BMP6, BMP2, PLAT, CD1D, PKD2, SERPINA1, PROCR, ALDH1A2*
Response to wounding	*TGFBR2, THBD, PTGER3, HBEGF, ACVRL1, CCL19, FOXF1, OSM, CD28, CD14, BMP6, BMP2, PLAT, SERPINA1, PROCR*
Regulation of leukocyte mediated immunity	*CD226, FOXF1, DPP4, CD28, CD1D, GIMAP1*
Pathway-restricted SMAD protein phosphorylation	*TGFBR2, ACVR1B, BMP6, BMP2*
Urogenital system development	*CRLF1, PTEN, CYP19A1, BMP6, BMP2, CA2, PKD2, ALDH1A2*
Vitamin metabolic process	*TKTL1, SLC22A4, CYP24A1, DHRS9, MTAP, PSAT1, ALDH1A2*
Positive regulation of alpha-beta T cell activation	*TGFBR2, CD28, CD1D, GIMAP1*
***KEGG pathway***	
TGF-beta signaling pathway	*TGFBR2, ACVRL1, ACVR1B, IDA, BMP6, BMP2*
***Drugs***	
Calcitriol	*EFTUD1, EHBP1, TRIM56, HBEGF, CYP24A1, CYP19A1, ACVRL1, ACVR1B, SEMAD6D, ARRDC4, CD14, BMP6, PRKD1, BMP2, CLMN, PLAT, IL1RL1, PRKCH, SLC1A1, CA2, FAM20C, SHE*

(Toppgene, p<0.05).

To determine overlapping genes up-regulated by both calcitriol concentrations (at FDR ≤ 0.1), a Venn diagram was assembled. This approach identified five commonly up-modulated genes: *CYP24A1, DPP4, EFTUD1, TKTL1* and *KCNK3*.

**Figure 2:**
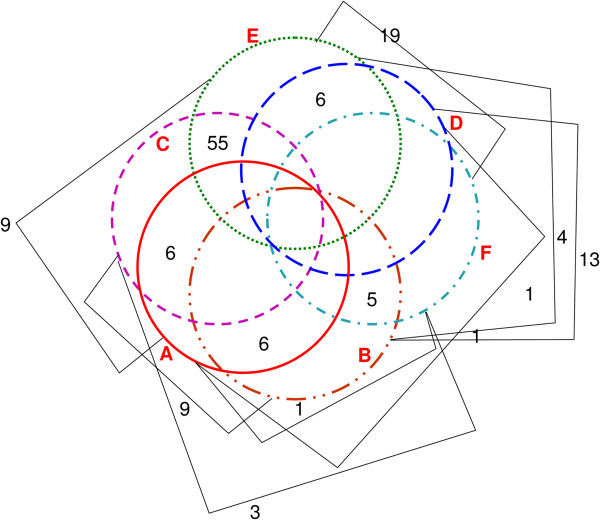
**Overlapping genes up-regulated by 1,25(OH)**_**2**_**D**_**3 **_**10-100nM in cell lines.** Venn diagram of 1,25(OH)_2_D_3_ up-regulated genes in breast cancer slices (**A**) cancer associated fibroblasts and normal adjacent fibroblasts (**B**) [40]; non-transformed prostate epithelial cell line (**C**) [44]; human coronary artery smooth muscle cells (**D**) [46]; SCC25 cells (floor of the mouth/base of the tongue squamous tumor, (**E**) [43] and lymphoblastoid cell lines (**F**) [45]. B, C, and D include non-transformed cells. Number of genes regulated in two cell lines appears on the diagram; number of genes regulated in more than two cell lines appears on the text.

The reproducibility of the present gene list was further tested against gene lists determined in other cell lines. To this end, vitamin D (100 nM) up-regulated genes were cross checked in breast cancer slices and derived fibroblasts [40], squamous carcinoma [43] immortalized prostate [44], and lymphoblastoid cell lines [45] as well as in carotid artery smooth muscle cells [46] (Figure 2). These cell lines were treated with supra-physiological concentrations, ranging from 10-100nM, of 1,25(OH)2D3 or EB1089 (vitamin D analog) for 12–36 hours. This analysis revealed *CYP24A1* as the universal vitamin D target gene in all cell types. Expression of *CLMN, EFTUD1* and *SERPINB1* was up-regulated in five of the six studies and *BMP6, CD14, FAM20C*, and *THBD* in four studies. *CA2, CILP, CYP19A1, DCBLD1, DPP4, FOXF1, G0S2, GRK5, IL1RL1, KCNK3, SEMA6D* and *SLC1A1* were up-regulated in another two studies, in addition to the present one. Many of these genes were also regulated by vitamin D in this organotypic culture.

A subset of seven genes was selected for further analysis in samples from another group of patients, using qPCR. Candidates were chosen from microarray analysis and included two genes modulated by both calcitriol concentrations: *CYP24A1* and *DPP4*; and five genes regulated by 100nM calcitriol at a fold change > 2, compared to control samples: *IL1RL1, SHE, CD14*, *CA2* and *BMP6*. Initially, significant correlations between gene expression values obtained from the microarray dataset and those obtained by subsequent qPCR analysis in the first group of five patients were evaluated, as a technical validation procedure. In these 15 samples (control; 0.5nM 1,25(OH)_2_D_3_ and 100nM 1,25(OH)_2_D_3_) significant direct correlations were demonstrated for all genes, except for *BMP6* (Additional file 5: Table S4).

**Figure 3:**
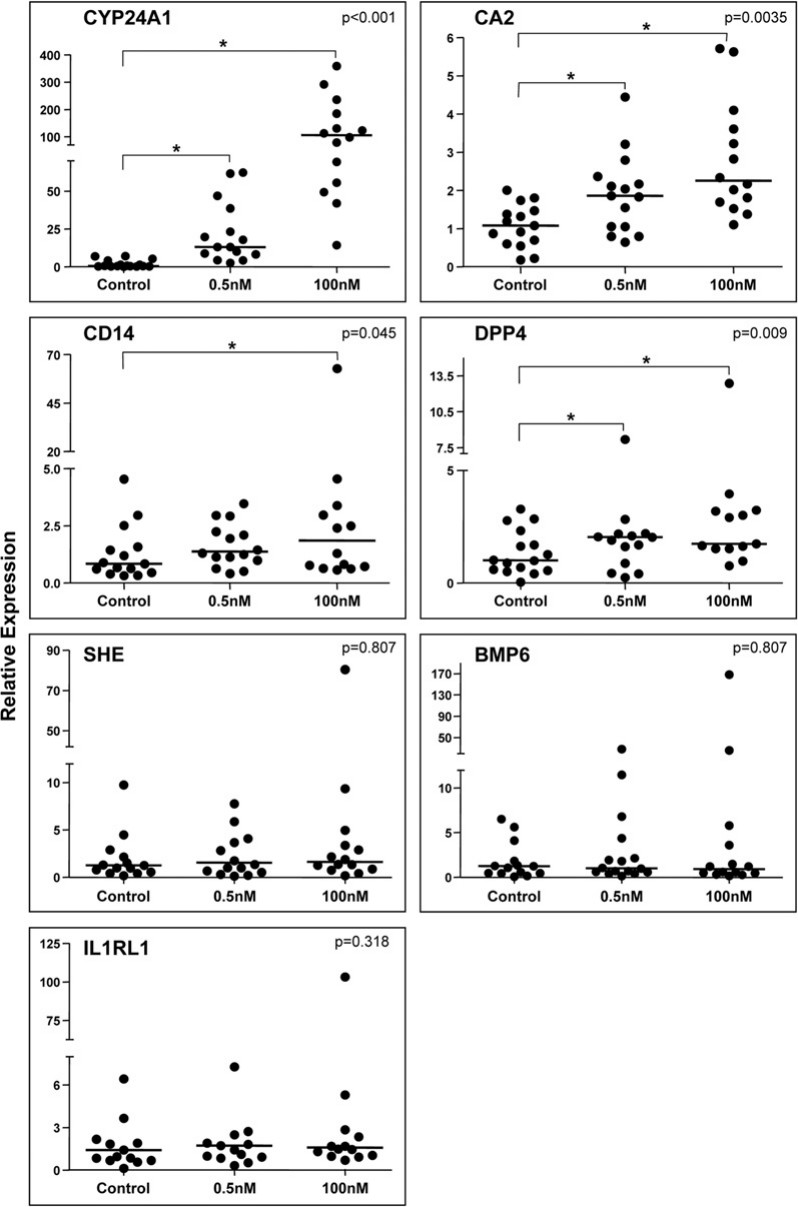
**Effect of 1,25(OH)**_**2**_**D**_**3 **_**on expression of *CYP24A1, CA2, CD14, DPP4, SHE, BMP6 and ILRL1 *in breast cancer slices.** Tumor specimens from 12–16 patients were treated or not (control) with 1,25(OH)_2_D_3_ (0.5nM or 100nM) for 24 hours and analyzed by qPCR. Relative expression was calculated as 2^-ΔΔCT^, using *GAPDH* as internal control and the average value of the target gene in control samples, as reference. Horizontal line represents the median value of gene expression. Friedman test, p value inside the box; * (over the horizontal bracket): p ≤ 0.05, Wilcoxon signed ranks test.

Subsequently, the expression of these seven genes was determined in samples from an additional group of 16 patients (validation subset). In these samples, *CYP24A1, DPP4* and *CA2* were up-regulated by both 1,25(OH)_2_D_3_ 0.5 and 100nM whereas CD14 expression was induced only by 1,25(OH)_2_D_3_ 100nM (Figure 3). Median expression of *IL1RL1, SHE*, and *BMP6* was not significantly up-regulated by either dose of 1,25(OH)_2_D_3_ in these additional samples, even though elevated mRNA levels were detected in a subset of tumors after treatment.

### Vitamin D transcriptional effects in epithelial and stromal cells

The effects of 1,25(OH)_2_D_3_ 0.5nM on the expression of *CYP24A1*, *DPP4, IL1RL1, CD14*, *CA2* and *BMP6,* were further explored in breast tumor derived cells, representing the epithelial and stromal compartments, using RT-qPCR. For this analysis, normal and cancerous breast cell lines (HB4A, C5.4, SKBR3, MDA-MB231, MCF-7) and cancer associated fibroblasts (primary cultures obtained from fresh tumor samples) were used. In the breast-derived epithelial cell lines, robust expression of *CYP24A1* was observed in all lineages, indicating functional VDR expression. Breast cell lines (HB4a, C5.4, SKBR3) that exhibited low baseline *CYP24A1* expression showed larger fold-induction of this gene than cell lines (MCF-7, MD-MBA-231) presenting high baseline *CYP24A1*. Expression of *CA2*, *CD14* and *IL1RL1*, was significantly induced by 1,25(OH)_2_D_3_ 0.5nM, but considerable variability in the response of individual lineages was observed, and cells displaying the most robust up-regulation of *CYP24A1* in response to 1,25(OH)_2_D_3_ did not necessarily exhibit the highest induction of the other target genes. Three of the breast cancer cell lines demonstrated up-regulation of *BMP6* in response to 1,25(OH)_2_D_3_ 0.5nM however, the group response was not statistically significant.

**Figure 4:**
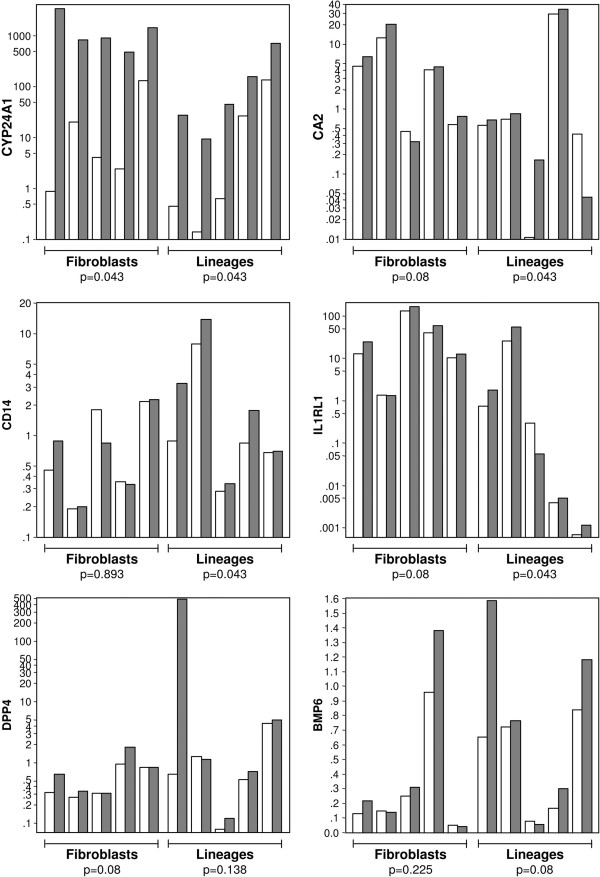
**Effect of 0.5nM 1,25(OH)**_**2**_**D**_**3 **_**on expression of *CYP24A1, CD14, CA2, DPP4, ILRL1 *and *BMP6 *in cancer infiltrating fibroblasts and mammary epithelial cells.** Cancer infiltrating fibroblasts (n=5) and breast derived cell lines (left to right: HB4A, C5.4, SKBR3, MCF7, MDA-MB231) were cultured in the presence or absence of 0.5nM 1,25(OH)_2_D_3_ for 24 h. Three independent assays for each cell line were performed and the mean relative gene expression value was calculated. Gene expression was determined by RT-qPCR and relative expression is shown on the Y axis (which is in log scale for *CYP24A1, CA2, CD14, DPP4 and IL1RL1*). p values: Wilcoxon signed ranks test. White bar: control samples; Gray bars: 1,25(OH)_2_D_3_ treated samples.

In five independently-derived primary cultures of cancer-associated fibroblasts, *CYP24A1* expression was consistently induced in response to 1,25(OH)_2_D_3_ 0.5nM indicating active VDR signaling in the tumor stroma. However, none of the other target genes, identified in the microarray analysis, were significantly up-regulated in tumor fibroblasts cultured with 0.5nM 1,25(OH)_2_D_3_*ex vivo* , even though there was a trend towards up-regulation of *CA2*, *IL1RL1* and *DPP4* (Figure 4).

### Vitamin D effects on protein expression

Vitamin D effects in protein expression were analyzed in tumor slices and culture medium, as well as in epithelial cell lines and fibroblasts.

**Figure 5:**
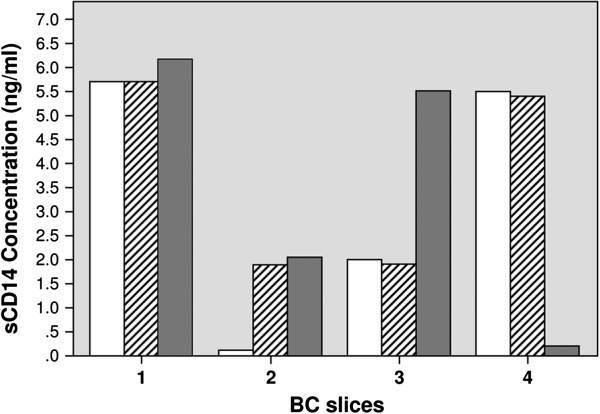
**CD14 expression in breast cancer samples.** Tumor slices were cultured in the absence (control: **B**, **E**) or presence of 1,25(OH)_2_D_3_ 100nM (**C**) or 0.5nM (**F**) for 24 h. Protein expression was examined by immunohistochemistry. CD14 expression was detected as a cytoplasmic staining. Negative reaction with secondary antibodies exclusively, appears on the left (**A**, **D**). scale bar: 25μm.

Tumor slices from seven patients (in FFPE, six samples cultured in the absence or presence of 1,25(OH)_2_D_3_ 100nM and one sample in the presence of 1,25(OH)_2_D_3_ 0.5nM) were available for immunohistochemistry. CD14 moderate cytoplasmic staining was observed in at least 50% of tumor cells and weak staining of 10% of the fibroblasts. No differences could be detected between 1,25(OH)_2_D_3_ treated and untreated tumor samples (Figure 5).

**Figure 6:**
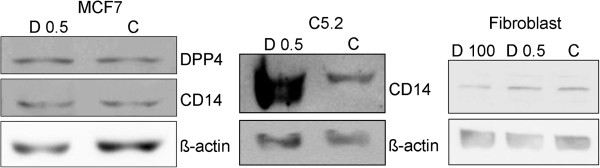
**Soluble CD14 in the conditioned medium of breast cancer slices.** Tumor specimens from four patients with invasive ductal carcinoma (not included in previous analysis of microarray or PCR) were cultured with or without (control, white bars) 1,25(OH)_2_D_3_ 0.5 (hatched bars) or 100 nM (gray bars) for 24 h. sCD14 concentration was evaluated in the conditioned medium by ELISA (p=0.42; Friedman’s test).

CD14 may be either soluble (sCD14) or membrane-bound (mCD14). There is evidence that sCD14 may be detected in plasma samples from breast cancer patients, hence we determined whether sCD14 concentration might be regulated in the culture medium of breast cancer slices. Although no significant statistical differences were found between control and calcitriol treated samples (0.5nM and 100nM), there was a trend towards higher values of sCD14 in 3/4 samples exposed to 1,25(OH)_2_D_3_ 100nM (Figure 6).

**Figure 7:**
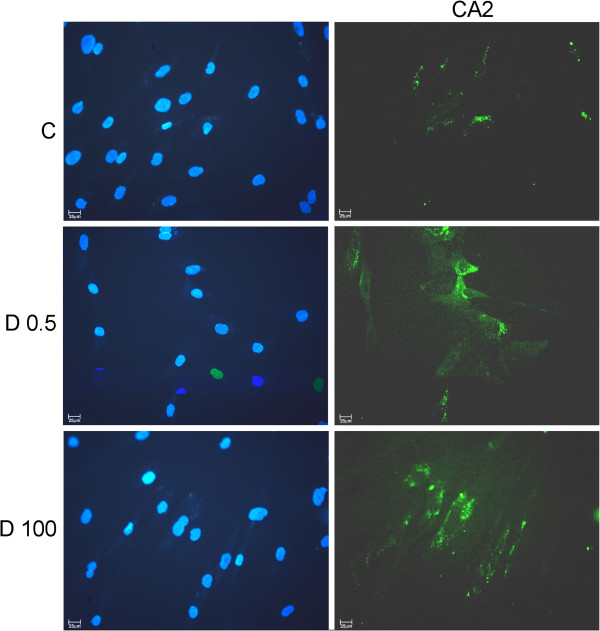
**CD14 and DPP4 (CD26) expression in mammary epithelial cell lines and fibroblasts.** MCF7 and C5.2 cells as well as fibroblasts were cultured in the absence (control, C) or presence of 1,25(OH)_2_D_3_ 0.5 nM (D 0.5). Protein expression was analyzed through western blot. Two assays were performed with similar results.

**Figure 8:**
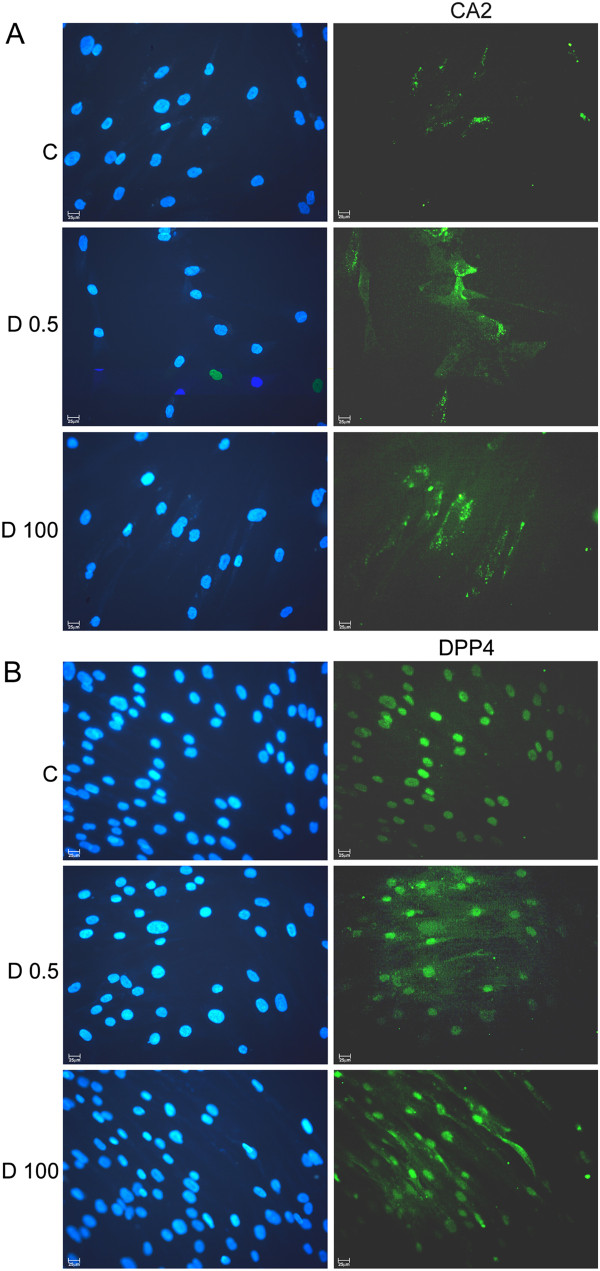
**CA2 and DPP4 (CD26) expression in fibroblasts.** Cancer associated fibroblasts from two patients with IDC were cultured in the presence or absence (control-1^st^ row) of 1,25(OH)_2_D_3_ 0.5 nM (2^nd^ row) or 100 nM (3^rd^ row) and processed for immunostaining with specific antibodies and DAPI for nuclear staining (on the left). (**A)** CA2 cytoplasmic granular staining and (**B)** DPP4 cytoplasmic staining appears in green. scale bar: 25μm.

In mammary epithelial cell lines and cancer associated fibroblasts protein expression was analyzed through western blot and immunocytochemistry. CD14 was more expressed in MCF7 and C5.2 treated with 0.5nM 1,25(OH)_2_D_3_, as compared with untreated cells, however no differences in CD14 expression were observed in fibroblasts exposed to 1,25(OH)_2_D_3_. In MCF7 cells, DPP4 (CD26) expression was also induced after 1,25(OH)_2_D_3_ 0.5nM exposure (Figure 7). In addition, in fibroblasts, CA2 as well as DPP4 cytoplasmic immunoexpression was more intense in 1,25(OH)_2_D_3_ treated than in control cells (Figure 8).

## Discussion

The primary goal of this work was to evaluate the transcriptional responses of breast cancer samples to physiologically relevant concentrations of 1,25(OH)_2_D_3_, using a culture model that retains features of intact tumors, such as stromal-epithelial interactions. Microarray analysis identified nine genes that were significantly altered within 24 h of exposure to 1,25(OH)_2_D_3_ 0.5nM, a concentration that is physiologically achievable in patients. Of these, the vitamin D target gene *CYP24A1*(which codes a cytochrome P450 enzyme, that hydroxylates 25(OH)D_3_ and 1,25(OH)_2_D_3_ to less active forms 24,25(OH)_2_D_3_ and 1,24,25(OH)_3_D_3_) was induced over 7-fold in microarray analysis and was validated in another set of tumor samples, clearly indicating activation of VDR signaling. Additional evidence for activation of the VDR pathway in this dataset was obtained by GSEA, which indicated a trend towards the enrichment of genes sharing DR3 binding sites, a consensus motif for VDR.

Comparison of microarray data from tumor slices cultured with 0.5nM vs. 100nM 1,25(OH)_2_D_3_ indicated a clear concentration effect, as the number of differentially expressed transcripts increased from nine at 0.5nM to 186 at 100nM (20 fold increment). Induction of CYP24A1 increased from 7-fold (at 0.5 nM) to 70-fold (at 100nM) - a 10 fold enhancement. In both datasets, the majority of genes (approximately 75%) were up-regulated rather than down-regulated by 1,25(OH)_2_D_3_, consistent with other array data from established cell lines cultured with high dose 1,25(OH)_2_D_3_*in vitro*[43-45].

In addition to *CYP24A1*, five other genes were commonly up-regulated in tumor slices exposed to both low and high concentrations of 1,25(OH)_2_D_3_: *DPP4, KCKN3, EFTUD1, TKTL1* and *CA2*. All, except *TKTL1* (transketolase-like 1) have been previously identified as VDR target genes in various model systems. *DPP4* (dipeptidyl-peptidase 4, also called CD26) was up-regulated in artery smooth muscle cells exposed to 1,25(OH)_2_D_3_[46] and its overexpression in distinct cell types (melanocytes, non-small cell lung, prostate and neuroblastoma cells) triggered anti-tumorigenic effects including cell growth arrest, inhibition of cell migration and increased apoptosis [47]. *KCNK3* (potassium channel, subfamily K, member) was induced by 1,25(OH)_2_D_3_ in artery smooth muscle cells, and *EFTUD1* (elongation factor Tu GTP binding domain containing 1) in oral squamous carcinoma, breast cancer associated fibroblasts, immortalized prostate cells and lymphoblastoid cell lines [40,43-46]. *CA2* (carbonic anhydrase II) mRNA appeared to be directly induced by 1,25(OH)_2_D_3_ in myelomonocytic cell lines but indirectly regulated in osteoclast progenitors, where the physical communication with stromal cells seems to be required [48,49]. *CYP26B1* (cytochrome P450, family 26, subfamily b, polypeptide 1) which was up-regulated in samples treated with 1,25(OH)_2_D_3_ 0.5nM, was previously identified as a vitamin D induced gene in immortalized non-transformed prostate epithelial and oral squamous carcinoma cell lines, and *in silico* analysis has tentatively identified a VDR binding site at this genomic region [43,44].

Other authors have analyzed physiological concentration effects of vitamin D using animal models. Vitamin D supplemented diet as well as calcitriol injections were shown to stimulate the VDR pathway, mildly increasing CYP24A1 expression (x2) in MCF-7 xenografts in immunocompromised mice [50]. Interestingly, vitamin D transcriptional effects may not overlap in tumor specimens and non-transformed mammary glands in the MMTV-neu transgenic mouse model of breast cancer, fed a high vitamin D diet [51]. Comparison between cancer and normal cells is an interesting issue, as vitamin D potential effects in cancer prevention have also been claimed. In accordance with the previous work [51], differences in transcriptional targets were also described for breast cancer associated fibroblasts (CAF) and normal adjacent fibroblasts (NAF) exposed to 1,25(OH)_2_D_3_ in a supra-physiological concentration. Among up-regulated genes 45.7% were commonly modulated in CAFs and NAFs, however, 36.4% were exclusively up-regulated in NAFs and 17.4% exclusively up-regulated in CAFs [40]. In addition, looking at overlapping genes in the Venn diagram of vitamin D up-regulated transcripts in six works [40,43-46], only seven intersections were found in non-cancer cells: *AKR1B1, CRIP1, FZD8, MREG* (in immortalized prostate cells and NAF), *BCAT1, GCLC* (in coronary artery smooth muscle cells and NAFs) and *PRR6* (in immortalized prostate cells and coronary artery smooth muscle cells). Furthermore, it was reported that vitamin D response is blunted in transformed HME normal mammary cells as compared with parental normal cells [52]. The last works evaluating vitamin D effects in normal cells however, were performed using supra-physiological concentrations of 1,25(OH)_2_D_3_ (10-100nM) or analogs and the role of physiological concentrations of the hormone in normal cells is not fully established.

At 100nM, 1,25(OH)_2_D_3_ exerted more extensive transcriptional effects, and at least 40 of the induced genes in breast cancer organotypic culture have already been reported as up regulated by the hormone, such as *ALCAM, ARRDC4, BMP2, BMP6, CA2, CD14, CLIC6, CILP, CLMN, CYP19A1, DCLDB1, EFTUD1, EHBP1, FAM20C, FOXF1, FRAS1, GOS2, GRK5, HBGEF, HSMPP8, IL1RL1, KCNK3, KIAA0500, PKD2, RGNEF, SEMA6D, SERPINB1, SLC1A1, THBD, TIMP1, TRIM56*[40,43-46]. However, co-aggregation of paired samples (treated and untreated) upon cluster analysis suggests that an individual dominant transcriptional profile was maintained, regardless of treatment. These results were not unexpected, as a high degree of transcriptional similarity was also demonstrated for matched pre and post-neoadjuvant chemotherapy, even though the chemotherapy exerts a more pronounced acute cellular effect than hormonal treatments [53-55].

Some of the genes induced by 100nM 1,25(OH)_2_D_3_ concentration are involved in TGF beta signaling pathway, in accordance with other authors [56,57]. Other genes are involved in regulation of leukocyte mediated immunity and positive regulation of alpha-beta T cell activation, including *CD14*, which encodes a receptor to bacterial lipopolysaccharide, as previously reported in a variety of cells as mononuclear phagocytes, normal human epidermal keratinocytes, oral squamous carcinoma, immortalized non-transformed prostate epithelial cell lines and malignant breast cells [43,56,58].

The present tumor slice model represents a heterogeneous combination of epithelial and stromal cells, in which the complex array of reciprocal interactions taking place in the tumor microenvironment, including cell-cell contacts and a variety of secreted factors, might modulate the overall response to 1,25(OH)_2_D_3_. Hence, after evaluating the hormone effects in tumor slices, the effects of 1,25(OH)_2_D_3_ 0.5nM in defined populations of cancer associated fibroblasts and epithelial cells were compared. This data indicated that even though *CYP24A1* was induced in both fibroblasts and epithelial cells, *CD14, CA2,* and *IL1RL1* were primarily induced in epithelial cells. There was also a trend towards up-regulation of *CA2, DPP4* and *IL1RL1* in cancer associated fibroblasts.

One major strengthen of this work was the comparison of achievable versus supra-physiological concentrations of 1,25(OH)_2_D_3_ in breast cancer slices, a model that preserves the epithelial-mesenchimal interactionss, indicating that effects are much less intense in near physiological concentrations. However, a weakness of this work was the small number of samples used in microarray experiments. These effects however, were later confirmed in a larger number of tumor samples and cell lines, using RT-PCR, even though they were more difficult to detect at the protein level, in face of the discrete changes induced by 0.5nM 1,25(OH)_2_D_3_.

## Conclusion

Our main conclusion is that a very modest transcriptional response may be observed after exposure to 1,25(OH)_2_D_3_, within the physiological concentration range. Gene targets in breast cancer samples, including *CYP24A1, DPP4* and *CA2,* seem to be shared by both fibroblasts and epithelial cells. A higher number of genes may be induced by a supra-physiological concentration of the hormone. Further studies employing physiological and supra-physiological concentrations may help to elucidate the hormone’s potential effects in breast cancer prevention and treatment, including calcitriol supplementation effects in post-menopausal women and calcitriol intra-tumoral effects in breast cancer xenografts.

## Competing interests

The authors declare that they have no competing interests.

## Authors’ contributions

CM designed the study, participated in data acquisition, participated in data analysis and interpretation, drafted and revised the manuscript; MLHK designed the study, participated in data analysis and interpretation, drafted and revised the manuscript; ECL, designed the study and participated in data acquisition; JW participated in data analysis and interpretation, drafted and revised the manuscript; LTC participated in data acquisition; MMB participated in data analysis and interpretation, drafted and revised the manuscript; MSM was responsible for the study in Hosp. A. C. Camargo and participated in data acquisition; RAR participated in data acquisition; PRV participated in data acquisition; JCGSG was responsible for the study in IBCC and participated in data acquisition; SN participated in data acquisition; RET participated in data acquisition; MAAKF designed the study, participated in data analysis and interpretation, drafted and revised the manuscript. All authors read and approved the final manuscript.

## Pre-publication history

The pre-publication history for this paper can be accessed here:

http://www.biomedcentral.com/1471-2407/13/119/prepub

## Supplementary Material

Additional file 1: Table S1 Primers. Click here for file

Additional file 2: Table S2 Gene sets enriched in breast tumor slices incubated in 0.5nM 1,25(OH)_2_D_3_. Click here for file

Additional file 3: Table S3 Genes differentially modulated in breast tumor slices incubated in 100 nM 1,25(OH)_2_D_3_. Click here for file

Additional file 4 **Unsupervised hierarchical clustering of breast cancer tissue slices exposed to vehicle or 1,25(OH)**_**2**_**D**_**3**_** 100 nM.** Click here for file

Additional file 5: Table 4 Correlation of gene expression values (microarray vs qPCR) evaluated in breast cancer slices. Click here for file
