# Supplementary material for: Transcriptional effects of 1,25 dihydroxyvitamin D3 physiological and supra-physiological concentrations in breast cancer organotypic culture
Source: BMC Cancer. 2013 Mar 15;13:119. doi: 10.1186/1471-2407-13-119 (PMC3637238; doi:10.1186/1471-2407-13-119)
Supplement: Additional file 1: Table S1 — Primers. [file 1471-2407-13-119-S1.doc]

### Supplementary Table 1. Primers.

| *Gene symbol* | *Genbank*  *accession n.* | *Primer Sequences* | *Amplicon*  *Size* | *Anneal.*  *Temp.* |
| --- | --- | --- | --- | --- |
| GAPDH* | NM_002046 | S 5' ATTCCACCCATGGCAAATTC 3' | 72bp | 58-60ºC |
|  |  | AS 5' TGATGGGATTTCCATTGATGA 3' |  |  |
|  |  | AS 5' GCCCACCATAAGACCTACGA 3' |  |  |
| CYP24A1 | NM_000181 | S 5' TATTTGCGGACAATCCAACA 3' | 249bp | 59-60ºC |
|  |  | AS 5' GGCAACAGTTCTGGGTGAAT 3' |  |  |
|  |  | AS 5' TGAAACACAGTTGGAGTGCAT 3' |  |  |
| BMP6 | NM_001718.4 | S 5' AAGGGCTATGCTGCCAATTA 3' | 114bp | 59-60ºC |
|  |  | AS 5' CTCGGGGTTCATAAGGTGAA 3' |  |  |
| CD14 | NM_000591.2 | S 5'GCTCAGAGGTTCGGAAGACT 3' | 115bp | 59-60ºC |
|  |  | AS 5' ATCGTCCAGCTCACAAGGTT 3' |  |  |
| IL1RL1 | NM_003856.2 | S 5' CTCATGTATTCCACAGCAGCA 3' | 111bp | 59-60ºC |
|  |  | AS 5'CCAATCCACGGTGTAACTAGG 3' |  |  |
| DPP4 | NM_001935.3 | S 5' GGTCGATGTTGGAGTGGATT 3' | 83bp | 59-60ºC |
|  |  | AS 5' TATGTTGGTGTGCTGTGCTG 3' |  |  |
| CA2 | NM_000067.2 | S 5' TCTGGAATGTGTGACCTGGA 3' | 122bp | 59-60ºC |
|  |  | AS 5' TGTCCACCATCAGTTCTTCG 3' |  |  |
| SHE | NM_001010846.1 | S 5' GCAGGTACTCCATTGCCCTA 3'  AS 5' TCAGGGATGCTGTCAAACAC 3' | 118bp | 59-60ºC |
