# Supplementary material for: Transcriptional effects of 1,25 dihydroxyvitamin D3 physiological and supra-physiological concentrations in breast cancer organotypic culture
Source: BMC Cancer. 2013 Mar 15;13:119. doi: 10.1186/1471-2407-13-119 (PMC3637238; doi:10.1186/1471-2407-13-119)
Supplement: Additional file 2: Table S2 — Gene sets enriched in breast tumor slices incubated in 0.5nM 1,25(OH)2D3. [file 1471-2407-13-119-S2.doc]

**Supplementary Table 2**. Gene sets enriched in calcitriol 0.5nM treated samples.

| Genes with promoter regions for VDR expressed in calcitriol 0.5nM phenotype |
| --- |
| CYP24A1, KCNK3, NBEA, ASXL2, SLC26A3, LIN28, HOXA13, ELAVL3, PITX2, DMD, SALL1, HOXB6, LRRC17, SLC27A3, CTNNBIA1, RBBP6, KCNC1, PRIC285, EPN1, BTF3L1, SLC25A28, RORC, DIAPH1, FLI1, TGFB3, ZBTB10, CTDSP1, PDGFB, GGTL3, ATP2A2, COL11A2, MAGEH1, RBM14, ERBB21P, HPCA, ZNF436 |

GSEA: motif, transcription factor. Permutation type: gene sets; FDR=0.098. Total number of genes in reference list: 104.
