# Supplementary material for: Transcriptional effects of 1,25 dihydroxyvitamin D3 physiological and supra-physiological concentrations in breast cancer organotypic culture
Source: BMC Cancer. 2013 Mar 15;13:119. doi: 10.1186/1471-2407-13-119 (PMC3637238; doi:10.1186/1471-2407-13-119)
Supplement: Additional file 3: Table S3 — Genes differentially modulated in breast tumor slices incubated in 100 nM 1,25(OH)2D3. [file 1471-2407-13-119-S3.doc]

**Supplementary Table 3**. Genes differentially modulated in calcitriol 100 nM treated tumor slices.

| *Gene title* | *Gene symbol* | *Fc* |
| --- | --- | --- |
| cytochrome P450, family 24, subfamily A, polypeptide 1 | **CYP24A1** | 71.21 |
| interleukin 1 receptor-like 1 | **IL1RL1** | 4.02 |
| cartilage intermediate layer protein, nucleotide pyrophosphohydrolase | **CILP** | 3.74 |
| potassium channel, subfamily K, member 3 | **KCNK3** | 3.67 |
| peptidase inhibitor 15 | PI15 | 3.60 |
| transmembrane protein 37 | **TMEM37** | 3.35 |
| Src homology 2 domain containing E | **SHE** | 3.22 |
| carbonic anhydrase II | CA2 | 3.20 |
| dipeptidyl-peptidase 4 (CD26, adenosine deaminase complexing protein 2) | **DPP4** | 2.58 |
| G0/G1switch 2 | **G0S2** | 2.51 |
| fructose-1,6-bisphosphatase 1 | FBP1 | 2.45 |
| forkhead box F1 | FOXF1 | 2.44 |
| transketolase-like 1 | **TKTL1** | 2.44 |
| CD300 molecule-like family member f | **CD300LF** | 2.41 |
| thrombomodulin | THBD | 2.35 |
| bone morphogenetic protein 6 | BMP6 | 2.34 |
| CDNA clone IMAGE:5299000 | NA | 2.34 |
| chimerin (chimaerin) 2 | **CHN2** | 2.32 |
| CD14 molecule | **CD14** | 2.26 |
| **CDNA FLJ41679 fis, clone HCASM2003212** | NA | 2.22 |
| sulfotransferase family, cytosolic, 1C, member 2 | **SULT1C**2 | 2.18 |
| carboxypeptidase M | **CPM** | 2.15 |
| CD226 molecule | **CD226** | 2.11 |
| purinergic receptor P2Y, G-protein coupled, 8 | P2RY8 | 2.08 |
| serpin peptidase inhibitor, clade B (ovalbumin), member 1 | **SERPINB1** | 2.08 |
| G protein-coupled receptor kinase 5 | **GRK5** | 2.07 |
| SRY (sex determining region Y)-box 7 | SOX7 | 2.06 |
| **CDNA FLJ37310 fis, clone BRAMY2016706** | NA | 2.04 |
| sema domain, transmembrane domain (TM), and cytoplasmic domain, (semaphorin) 6D | SEMA6D | 2.03 |
| family with sequence similarity 20, member A | FAM20A | 1.98 |
| elongation factor Tu GTP binding domain containing 1 | **EFTUD1** | 1.93 |
| dehydrogenase/reductase (SDR family) member 9 | DHRS9 | 1.92 |
| palmdelphin | PALMD | 1.89 |
| oncostatin M | OSM | 1.87 |
| Hypothetical protein LOC339524 | LOC339524 | 1.85 |
| MRNA; cDNA DKFZp313B1017 (from clone DKFZp313B1017) | NA | 1.84 |
| CD28 molecule | CD28 | 1.81 |
| Scavenger receptor class A, member 5 (putative) | SCARA5 | 1.78 |
| activin A receptor type II-like 1 | ACVRL1 | 1.76 |
| Fraser syndrome 1 | FRAS1 | 1.76 |
| vitelline membrane outer layer 1 homolog (chicken) | **VMO1** | 1.76 |
| amyloid beta (A4) precursor protein-binding, family B, member 1 interacting protein | APBB1IP | 1.75 |
| calmin (calponin-like, transmembrane) | **CLMN** | 1.72 |
| GTPase, IMAP family member 1 | GIMAP1 | 1.71 |
| KIAA0500 protein | **KIAA0500** | 1.71 |
| fibrinogen-like 2 | FGL2 | 1.70 |
| aldehyde dehydrogenase 1 family, member A2 | ALDH1A2 | 1.69 |
| CD1d molecule | **CD1D** | 1.69 |
| tripartite motif-containing 35 | TRIM35 | 1.68 |
| GTPase, IMAP family member 4 | GIMAP4 | 1.67 |
| ADAM-like, decysin 1 | ADAMDEC1 | 1.67 |
| cytochrome P450, family 19, subfamily A, polypeptide 1 | CYP19A1 | 1.67 |
| Family with sequence similarity 124A | FAM124A | 1.65 |
| **CDNA FLJ32207 fis, clone PLACE6003204** | NA | 1.65 |
| STEAP family member 3 | STEAP3 | 1.65 |
| Placenta mRNA, clone PL45, partial sequence | NA | 1.65 |
| GTPase, IMAP family member 6 | GIMAP6 | 1.65 |
| chloride intracellular channel 6’ | **CLIC6** | 1.64 |
| TIMP metallopeptidase inhibitor 1 | TIMP1 | 1.63 |
| Parathyroid hormone-like peptide mRNA, 3' end | NA | 1.63 |
| Homo sapiens, clone IMAGE:5180231, mRNA | NA | 1.60 |
| coenzyme Q2 homolog, prenyltransferase (yeast) | **COQ2** | 1.60 |
| zinc finger protein 709 | ZNF709 | 1.58 |
| G protein-coupled receptor 171 | GPR171 | 1.58 |
| RCSD domain containing 1 | **RCSD1** | 1.56 |
| latexin | LXN | 1.55 |
| phosphatase and tensin homolog (mutated in multiple advanced cancers 1) | **PTEN** | 1.54 |
| damage-regulated autophagy modulator | DRAM | 1.54 |
| hypothetical protein LOC153346 | LOC153346 | 1.54 |
| protein C receptor, endothelial (EPCR) | PROCR | 1.54 |
| family with sequence similarity 20, member C | FAM20C | 1.54 |
| docking protein 5 | DOK5 | 1.52 |
| protein phosphatase 1, regulatory (inhibitor) subunit 16B | PPP1R16B | 1.52 |
| caspase recruitment domain family, member 6 | CARD6 | 1.51 |
| ATP-binding cassette, sub-family B (MDR/TAP), member 4 | ABCB4 | 1.51 |
| hypothetical protein LOC134466 | LOC134466 | 1.50 |
| plasminogen activator, tissue | PLAT | 1.49 |
| THAP domain containing, apoptosis associated protein 2 | THAP2 | 1.49 |
| frequently rearranged in advanced T-cell lymphomas | FRAT1 | 1.49 |
| chemokine (C-C motif) ligand 19 | CCL19 | 1.49 |
| stimulated by retinoic acid gene 6 homolog (mouse) | STRA6 | 1.49 |
| lymphocyte cytosolic protein 2 (SH2 domain containing leukocyte protein of 76kDa) | LCP2 | 1.48 |
| family with sequence similarity 78, member A | FAM78A | 1.48 |
| discoidin, CUB and LCCL domain containing 1 | DCBLD1 | 1.48 |
| spondin 1, extracellular matrix protein | SPON1 | 1.48 |
| bone morphogenetic protein 2 | BMP2 | 1.47 |
| Clone 25015 mRNA sequence | NA | 1.47 |
| DnaJ (Hsp40) homolog, subfamily C, member 16 | DNAJC16 | 1.47 |
| Inhibitor of DNA binding 4, dominant negative helix-loop-helix protein | ID4 | 1.47 |
| transforming growth factor, beta receptor II (70/80kDa) | TGFBR2 | 1.47 |
| Na+/H+ exchanger domain containing 2 | NHEDC2 | 1.46 |
| Serpin peptidase inhibitor, clade A (alpha-1 antiproteinase, antitrypsin), member 1 | SERPINA1 | 1.45 |
| Full length insert cDNA clone ZD66F04 | NA | 1.45 |
| leptin receptor overlapping transcript-like 1 | LEPROTL1 | 1.44 |
| chromosome 2 open reading frame 32 | C2orf32 | 1.43 |
| SET domain and mariner transposase fusion gene | SETMAR | 1.43 |
| Rho-guanine nucleotide exchange factor | RGNEF | 1.43 |
| protein kinase D1 | PRKD1 | 1.42 |
| activin A receptor, type IB | ACVR1B | 1.42 |
| polycystic kidney disease 2 (autosomal dominant) | PKD2 | 1.42 |
| M-phase phosphoprotein, mpp8 | HSMPP8 | 1.41 |
| protein kinase C, eta | PRKCH | 1.41 |
| MRNA; cDNA DKFZp762N156 (from clone DKFZp762N156) | NA | 1.40 |
| phospholipase C-like 2 | PLCL2 | 1.40 |
| armadillo repeat containing 8 | ARMC8 | 1.40 |
| inhibitor of growth family, member 3 | ING3 | 1.40 |
| prostaglandin E receptor 3 (subtype EP3) | PTGER3 | 1.39 |
| WD repeat domain 43 | WDR43 | 1.39 |
| stonin 2 | STON2 | 1.38 |
| EF-hand calcium binding domain 2 | EFCAB2 | 1.38 |
| solute carrier family 1 (neuronal/epithelial high affinity glutamate transporter, system Xag), member 1 | SLC1A1 | 1.38 |
| XPA binding protein 1, GTPase | XAB1 | 1.37 |
| solute carrier family 22 (organic cation transporter), member 4 | SLC22A4 | 1.36 |
| CDNA FLJ12304 fis, clone MAMMA1001878 | NA | 1.35 |
| chromosome 9 open reading frame 125 | C9orf125 | 1.35 |
| cytokine receptor-like factor 1 | CRLF1 | 1.34 |
| NLR family, CARD domain containing 5 | NLRC5 | 1.34 |
| hypothetical LOC387723 | hypothetical protein LOC651940 | LOC387723 | 1.34 |
| epidermal growth factor receptor pathway substrate 8 | EPS8 | 1.34 |
| ELISC-1 | NA | 1.33 |
| development and differentiation enhancing factor 2 | DDEF2 | 1.32 |
| transducin-like enhancer of split 4 (E(sp1) homolog, Drosophila) | TLE4 | 1.32 |
| phosphoserine aminotransferase 1 | PSAT1 | 1.32 |
| tripartite motif-containing 56 | TRIM56 | 1.31 |
| Two transmembrane domain family member A | TTMA | 1.31 |
| heparin-binding EGF-like growth factor | HBEGF | 1.31 |
| CDNA clone IMAGE:4791585 | NA | 1.31 |
| coiled-coil domain containing 92 | CCDC92 | 1.31 |
| methylthioadenosine phosphorylase | MTAP | 1.30 |
| arrestin domain containing 4 | ARRDC4 | 1.30 |
| EH domain binding protein 1 | EHBP1 | 1.30 |
| similar to hypothetical protein 9630041N07 | DKFZp686E2433 | 1.29 |
| PDZ and LIM domain 4 | PDLIM4 | 1.28 |
| MIS12, MIND kinetochore complex component, homolog (yeast) | MIS12 | 1.26 |
| adaptor-related protein complex 3, mu 1 subunit | AP3M1 | 1.25 |
| FYN oncogene related to SRC, FGR, YES | FYN | 1.18 |
| oxidation resistance 1 | OXR1 | -1.26 |
| S-adenosylhomocysteine hydrolase-like 1 | AHCYL1 | -1.27 |
| growth factor receptor-bound protein 2 | GRB2 | -1.30 |
| inositol monophosphatase domain containing 1 | IMPAD1 | -1.30 |
| UDP-glucose ceramide glucosyltransferase | UGCG | -1.30 |
| CDNA FLJ39726 fis, clone SMINT2015306 | NA | -1.31 |
| PHD finger protein 20-like 1 | PHF20L1 | -1.31 |
| FERM domain containing 6 | FRMD6 | -1.31 |
| DIS3 mitotic control homolog (S. cerevisiae)-like 2 | DIS3L2 | -1.35 |
| ADP-ribosylation factor guanine nucleotide-exchange factor 2 (brefeldin A-inhibited) | ARFGEF2 | -1.36 |
| ankyrin repeat domain 44 | ANKRD44 | -1.36 |
| protein tyrosine phosphatase, receptor type, f polypeptide (PTPRF), interacting protein (liprin), alpha 1 | PPFIA1 | -1.37 |
| casein kinase 2, alpha 1 polypeptide | CSNK2A1 | -1.38 |
| calcium/calmodulin-dependent protein kinase ID | CAMK1D | -1.39 |
| Rho-related BTB domain containing 3 | RHOBTB3 | -1.39 |
| Zinc finger protein 24 | ZNF24 | -1.40 |
| SNF1-like kinase | SNF1LK | -1.41 |
| adenylate kinase 3-like 1 | adenylate kinase 3-like 2 | similar to Adenylate kinase isoenzyme 4, mitochondrial (ATP-AMP transphosphorylase) | AK3L1 | -1.42 |
| zinc finger protein, X-linked | ZFX | -1.42 |
| WD repeat domain 59 | WDR59 | -1.43 |
| solute carrier family 2 (facilitated glucose transporter), member 3 | SLC2A3 | -1.43 |
| PTC7 protein phosphatase homolog (S. cerevisiae) | PPTC7 | -1.43 |
| Folliculin interacting protein 1 | FNIP1 | -1.44 |
| NECAP endocytosis associated 2 | NECAP2 | -1.44 |
| baculoviral IAP repeat-containing 4 | BIRC4 | -1.45 |
| OCIA domain containing 1 | OCIAD1 | -1.45 |
| Full length insert cDNA clone ZC66E08 | NA | -1.47 |
| mitogen-activated protein kinase kinase kinase 2 | MAP3K2 | -1.47 |
| SMEK homolog 2, suppressor of mek1 (Dictyostelium) | SMEK2 | -1.48 |
| pleckstrin homology-like domain, family B, member 2 | PHLDB2 | -1.49 |
| toll-like receptor 2 | TLR2 | -1.51 |
| neuronal pentraxin I | NPTX1 | -1.51 |
| CUB domain containing protein 1 | CDCP1 | -1.51 |
| zinc finger, MYM-type 2 | ZMYM2 | -1.52 |
| Transcribed locus | NA | -1.52 |
| YME1-like 1 (S. cerevisiae) | YME1L1 | -1.53 |
| cyclin T1 | CCNT1 | -1.55 |
| adult retina protein | LOC153222 | -1.56 |
| PTK2 protein tyrosine kinase 2 | PTK2 | -1.58 |
| phosphoinositide-3-kinase, class 2, alpha polypeptide | PIK3C2A | -1.58 |
| superoxide dismutase 2, mitochondrial | SOD2 | -1.58 |
| guanine nucleotide binding protein (G protein), alpha 13 | GNA13 | -1.58 |
| Splicing factor, arginine/serine-rich 11 | SFRS11 | -1.58 |
| vimentin | VIM | -1.58 |
| interferon induced with helicase C domain 1 | IFIH1 | -1.58 |
| cortactin | CTTN | -1.59 |
| myosin, heavy chain 10, non-muscle | MYH10 | -1.61 |
| triple functional domain (PTPRF interacting) | TRIO | -1.62 |
| Period homolog 3 (Drosophila) | PER3 | -1.64 |
| hairy and enhancer of split 4 (Drosophila) | HES4 | -1.64 |
| Zinc finger and BTB domain containing 11 | ZBTB11 | -1.67 |
| phosphoglycerate kinase 1 | **PGK1** | -1.68 |
| Small nuclear ribonucleoprotein polypeptide A' | SNRPA1 | -1.68 |
| nucleoporin like 1 | **NUPL1** | -1.68 |
| nuclear receptor subfamily 4, group A, member 3 | NR4A3 | -1.72 |
| Squalene epoxidase | SQLE | -1.76 |
| activated leukocyte cell adhesion molecule | ALCAM | -1.79 |
| reticulon 4 | RTN4 | -1.86 |
| purinergic receptor P2Y, G-protein coupled, 1 | P2RY1 | -2.15 |
| BCL6 co-repressor | BCOR | -2.75 |

SAM paired analysis FDR ≤ 0.10 (FDR ≤ 0.01 marked in bold). Fc: Fold change of the ratio. (-): less expressed in calcitriol 100nM treated (as compared with untreated) samples.
