# Supplementary figures and images for: Transcriptional effects of 1,25 dihydroxyvitamin D3 physiological and supra-physiological concentrations in breast cancer organotypic culture
Source: BMC Cancer. 2013 Mar 15;13:119. doi: 10.1186/1471-2407-13-119 (PMC3637238; doi:10.1186/1471-2407-13-119)

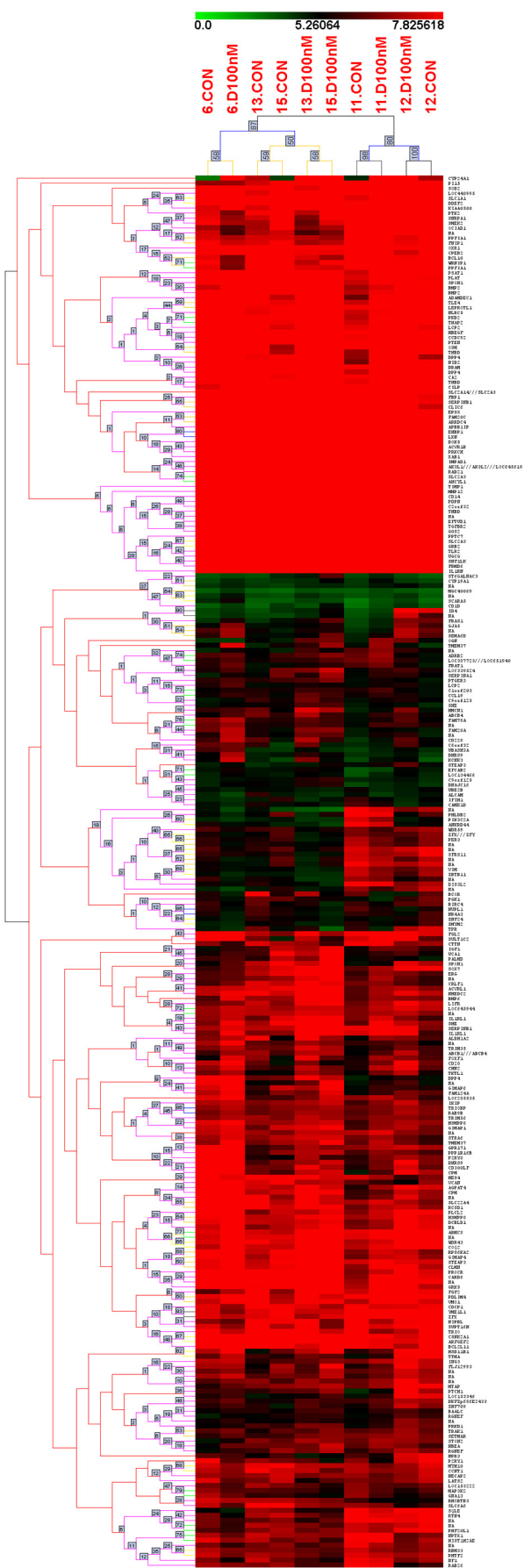

Supplement: Additional file 4 — Unsupervised hierarchical clustering of breast cancer tissue slices exposed to vehicle or 1,25(OH)2D3 100 nM. [file 1471-2407-13-119-S4.pdf]
