# Supplementary material for: Transcriptional effects of 1,25 dihydroxyvitamin D3 physiological and supra-physiological concentrations in breast cancer organotypic culture
Source: BMC Cancer. 2013 Mar 15;13:119. doi: 10.1186/1471-2407-13-119 (PMC3637238; doi:10.1186/1471-2407-13-119)
Supplement: Additional file 5: Table 4 — Correlation of gene expression values (microarray vs qPCR) evaluated in breast cancer slices. [file 1471-2407-13-119-S5.doc]

**Supplementary Table 4**. Correlation of gene expression values (microarray vs qPCR) evaluated in breast cancer slices.

| ***Gene symbol*** | ***Gene expression values (microarray vs qPCR)*** | |
| --- | --- | --- |
|  | **r** | **p** |
| CD14 | 0.682 | 0.005^α^ |
| DPP4 | 0.952 | <0.001^β^ |
| BMP6 | 0.029 | 0.919^β^ |
| CYP24A1 | 0.907 | <0.001^β^ |
| IL1RL1 | 0.807 | <0.001^β^ |
| CA2 | 0.954 | <0.001^β^ |
| SHE | 0.804 | <0.001^β^ |

Gene expression values were evaluated in 15 samples from 5 patients: control (n=5); calcitriol 0.5nM (n=5); calcitriol 100nM (n=5) by microarray and qPCR. Kolmogorov-Smirnov was employed to test for normality of values followed by Pearson (α) or Spearman (β) correlation tests, as appropriate. r: correlation coefficient.
